# Grey Matter Volumes in Children with Conduct Problems and Varying Levels of Callous-Unemotional Traits

**DOI:** 10.1007/s10802-015-0073-0

**Published:** 2015-09-14

**Authors:** Catherine L. Sebastian, Stéphane A. De Brito, Eamon J. McCrory, Zoe H. Hyde, Patricia L. Lockwood, Charlotte A.M. Cecil, Essi Viding

**Affiliations:** Department of Psychology, Royal Holloway, University of London, London, UK; School of Psychology, University of Birmingham, Birmingham, UK; Division of Psychology and Language Sciences, University College London, London, UK; Department of Psychology, Division of Psychology and Language Sciences, University College London, 26 Bedford Way, London, WC1H 0AP UK

**Keywords:** Conduct problems, Conduct disorder, Callous-unemotional traits, Voxel-based morphometry, Grey matter volume, Orbitofrontal cortex

## Abstract

Genetic, behavioural and functional neuroimaging studies have revealed that different vulnerabilities characterise children with conduct problems and high levels of callous-unemotional traits (CP/HCU) compared with children with conduct problems and low callous-unemotional traits (CP/LCU). We used voxel-based morphometry to study grey matter volume (GMV) in 89 male participants (aged 10–16), 60 of whom exhibited CP. The CP group was subdivided into CP/HCU (*n* = 29) and CP/LCU (*n* = 31). Whole-brain and regional GMV were compared across groups (CP vs. typically developing (TD) controls (*n* = 29); and CP/HCU vs. CP/LCU vs. TD). Whole-brain analyses showed reduced GMV in left middle frontal gyrus in the CP/HCU group compared with TD controls. Region-of-interest analyses showed reduced volume in bilateral orbitofrontal cortex (OFC) in the CP group as a whole compared with TD controls. Reduced volume in left OFC was found to be driven by the CP/HCU group only, with significant reductions relative to both TD controls and the CP/LCU group, and no difference between these latter two groups. Within the CP group left OFC volume was significantly predicted by CU traits, but not conduct disorder symptoms. Reduced right anterior cingulate cortex volume was also found in CP/HCU compared with TD controls. Our results support previous findings indicating that GMV differences in brain regions central to decision-making and empathy are implicated in CP. However, they extend these data to suggest that some of these differences might specifically characterise the subgroup with CP/HCU, with GMV reduction in left OFC differentiating children with CP/HCU from those with CP/LCU.

**Electronic supplementary material:**

The online version of this article (doi:10.1007/s10802-015-0073-0) contains supplementary material, which is available to authorized users.

Children and adolescents with conduct disorder (CD) and conduct problems (CP) engage in high levels of antisocial and aggressive behaviour, and represent a significant public health burden (Erskine et al. 2014). CP is a heterogeneous category, and one fruitful approach has distinguished between CP children presenting with high (CP/HCU) and low (CP/LCU) levels of callous-unemotional (CU) traits. CU traits index low levels of empathy and guilt, a tendency to use and manipulate others, unconcern about achievement, and flattened emotional responsivity (Essau et al. 2006). Children with CP/HCU represent a particularly severe subgroup within CP (Frick and Viding 2009). Genetic, behavioural, cognitive and functional neuroimaging studies have shown that different vulnerabilities characterise these two subgroups of children with CP. However, little previous structural neuroimaging work has directly compared these groups. The current study used voxel-based morphometry with a large sample (N = 89) to study grey matter (GM) volumes in these two groups relative to both each other and to typically developing (TD) controls.

Evidence suggests that underlying aetiology and neurocognitive processing differ between CP/HCU and CP/LCU. Twin studies have shown that antisocial behaviour is more strongly heritable in children with CP/HCU than CP/LCU (Viding et al. 2005; Viding et al. 2008). Behavioural studies have also shown that CP/HCU is associated with a distinctive information processing profile relative to TD controls, most notably low reactivity to emotional cues (Blair et al. 2001), poor empathy (Jones et al. 2010), impulsivity (Fanti 2013), and poor reversal learning (Budhani and Blair 2005). This profile is similar to that shown by adults with psychopathy (Barry et al. 2000). In contrast, children with CP/LCU show increased emotional reactivity and a profile of reactive aggression and poor emotion regulation, with affective empathy relatively intact (Dadds et al. 2006; De Wied et al. 2012; Eisenberg et al. 2010; Frick et al. 2003; Jones et al. 2010).

Current theories regarding the neurobiology of psychopathic and CU traits suggest that affective and reinforcement learning deficits are underpinned by atypical function in a ventromedial prefrontal cortex-amygdala circuit (Blair et al. 2014) as well as in a more distributed paralimbic network including orbitofrontal cortex (OFC), anterior insula (AI), anterior and posterior cingulate, temporal pole and parahippocampal gyrus (Anderson and Kiehl 2012). Functional magnetic resonance imaging (fMRI) studies in children with CP/HCU have found largely reduced responsiveness across this network relative to TD controls during a variety of emotion processing (Jones et al. 2009; Lockwood et al. 2013; Marsh and Blair 2008; Sebastian et al. 2012) and decision-making (Finger et al. 2011; Marsh et al. 2011) tasks, although some studies have also found increased responses (Cohn et al. 2013). In contrast, fMRI studies which have looked at both CP/HCU and CP/LCU within the same study have found a different pattern of neural response in CP/LCU, for example increased (as opposed to decreased) amygdala response to emotional faces compared with TD controls (Sebastian et al. 2014; Viding et al. 2012).

However, to date, no study has directly compared brain structure in children with CP/HCU, CP/LCU and TD controls. Several structural MRI (sMRI) studies have compared children and adolescents with CP in general against TD controls. The most common technique has been to use voxel-based morphometry (VBM) to explore grey matter volume and/or concentration across the whole brain and within specific regions of interest (ROIs). These studies have found GM reductions in CP relative to TD controls in the amygdala (Cope et al. 2014a; Fairchild et al. 2011; Huebner et al. 2008; Sterzer et al. 2007) AI (Fairchild et al. 2011; Sterzer et al. 2007) OFC (Cope et al. 2014a), and temporal poles (Huebner et al. 2008). Findings in the amygdala in female participants have been similar (Fairchild et al. 2013). Use of alternative metrics such as cortical thickness has also shown reductions in cingulate, prefrontal and insular cortices (Fahim et al. 2011) and in several temporal and parietal regions (Hyatt et al. 2012; Wallace et al. 2014). The study by Wallace and colleagues also found some evidence of reduced gyrification in ventromedial prefrontal cortex and a significant negative correlation between the severity of CU traits and cortical thickness in the right superior temporal cortex. Overall, therefore, most studies show a general pattern of reduced GM across children and young people with CP in brain regions associated with antisocial behaviour and psychopathy, even after controlling for common confounding variables such as IQ, substance misuse and attention deficit hyperactivity disorder (ADHD) symptoms.

It is worth noting, however, that one previous study from our laboratory investigating a subclinical sample of boys with CP/HCU compared with TD controls found increased GM concentrations in medial OFC and anterior cingulate cortex (ACC), and increased grey matter volume and concentration in the temporal lobes bilaterally (De Brito et al. 2009). Unfortunately, in the absence of the CP/LCU group it was not possible to determine whether this pattern of findings was unique to CP/HCU. In addition, given the young age of the participants (mean age 11 years 8 months) it is not clear whether the pattern of increased GM concentration generalises to the broader CP population, in particular to older adolescents with CP. The lack of a developmentally typical reduction in GM concentration in key ROIs with increasing age in the CP/HCU group was responsible for the group difference seen in this study. The result was therefore interpreted as reflecting delayed cortical maturation in the CP/HCU sample. This pattern may not be evident if a CP population is sampled at a later age.

Of the sMRI studies discussed above, a subset have explored the contribution of CU or psychopathic traits to variance in grey matter indices across the CP sample. The pattern of findings has been somewhat mixed. For example, in a very large (N = 191) sample of incarcerated male adolescents (Ermer et al. 2013) found negative associations between GM volume and psychopathic traits in OFC extending into temporal poles and parahippocampal cortex, and posterior cingulate cortex. This pattern of results was recently replicated in a female incarcerated sample (Cope et al. 2014b). However, also in female participants, Fairchild et al. (2013) found that CU traits were positively (rather than negatively) associated with bilateral OFC volumes. Furthermore, some studies have found no associations between brain structure and CU traits; for example Fairchild et al. (2011) found no relationship between GM volumes and CU traits in males, although CD symptoms were negatively associated with right insula volume and the CD sample size was large (N = 63).

Given this mixed pattern of findings, we focused on whether males with CP/HCU and CP/LCU show similar structural profiles at the neural level, or whether certain structural deficits may characterise one or the other subgroup. Given that CP/HCU and CP/LCU show different behavioural profiles and genetic and neurocognitive vulnerabilities, we predicted that differences might also extend to the structural level. We focused on four regions of interest previously associated with atypical structure and function in CP/HCU and adult psychopathy (OFC, amygdala, AI and ACC), and predicted that structural differences in at least some of these regions would be driven by the CP/HCU group, with CP/LCU not differing from TD controls. However, since our previous study (De Brito et al. 2009) found increased GM while other studies have found reduced GM in this population, we did not predict the direction of effects a priori. We divided a large sample (N = 60) of children with CP into CP/HCU and CP/LCU subgroups. We compared grey matter volumes at the whole brain level and in ROIs using DARTEL-based VBM, both across CP participants relative to TD controls, and between all three groups. We also explored continuous relationships between GM volumes and both CU traits and conduct disorder symptoms across the sample of children with CP.

## Material and Methods

### Participants

Participants overlapped with a sample reported previously (Lockwood et al. 2013; Sebastian et al. 2014; Sebastian et al. 2012; Viding et al. 2012) although none of the data reported here have been published previously. Participants were recruited via local schools and advertisements; full details of sample recruitment are reported in these studies. Participant characteristics are displayed in Table 1. All aspects of the study were approved by the University College London Research Ethics Committee (Project ID number: 0622/001) and work was conducted in accordance with the Declaration of Helsinki. Informed consent was given by parents/guardians, while informed assent was given by participants themselves.

**Table 1:** Demographic data. In all columns, numbers in parentheses show standard deviation and [range of scores]

	Group				
Characteristics and questionnaires	TD Controls^1^ (*n* = 29)	CP/LCU^2^ (*n* = 31)	CP/HCU^3^ (*n* = 29)	*P* value	Post-hoc*
Age^b^	13.60 (1.53) [11.04–16.79]	14.16 (1.58) [10.77–16.90]	14.35 (1.64) [10.22–16.90]	0.18	
Socio-Economic Status^b^	2.93 (1.00) [1.67–5]	2.80 (1.22) [1–5]	3.34 (1.22) [1–5]	0.18	
Full IQ score from 2-subtest WASI^c^	105.21 (11.94) [74–129]	104.07 (11.53) [86–154]	97.97 (13.84) [73–126]	0.06	
Ethnicity^b^^,^^e^	23:2:3:1	20:4:5:2	22:2:5:0	0.72	
Handedness^b^^,^^f^	24:4:1	28:3:0	26:3:0	0.66	
Inventory of Callous-Unemotional Traits^d^	25.17 (4.94) [15–36]	34.97 (6.60) [15–42]	52.45 (6.56) [43–65]	0	1 < 2 < 3
Child and Adolescent Symptom Inventory					
Conduct Disorder^d^	0.55 (0.74) [0–2]	7.33 (2.73) [3–14]	13.76 (7.22) [5–30]	<0.001	1 < 2 < 3
Attention Deficit Hyperactivity Disorder^g^^,^^h^	9.70 (6.58) [0–27]	20.68 (11.07) [4–41]	31.20 (10.03) [12–49]	<0.001	1 < 2 < 3
Generalised Anxiety Disorder^g^^,^^h^	3.06 (3.03) [0–11]	6.59 (4.49) [0–20]	8.71(5.54) [1–21.71]	<0.001	1 < 2/3
Major Depressive Episode^g^^,^^i^	2.66 (1.54) [2–10]	5.48 (3.60) [2–15]	6.33 (4.74) [2–20]	<0.001	1 < 2/3
Alcohol Use and Disorders^c^^,^^h^	1.07 (1.49) [0–6]	2.50 (4.46) [0–21]	4.28 (6.59) [0–25]	0.04	1 < 3
Drug Use and Disorders^c^^,^^h^	0.10 (0.41) [0–2]	2.33 (4.79) [0–21]	2.24 (6.70) [0–34]	0.14	

*WASI* Wechsler abbreviated scale of intelligence, *TD* typically developing, *CP/LCU* conduct problems and low levels of callous–unemotional traits, *CP/HCU* conduct problems and high levels of callous–unemotional traits

*p < 0.05, Bonferroni corrected

^a^All *p*-values obtained using analysis of variance except for Ethnicity and Handedness (Fisher’s exact tests used)

^b^Measures taken at screening phase, parent report

^c^Measure completed by child at scanning session

^d^Measures taken at screening phase, parent and teacher report

^e^White:Black:Mixed:Asian

^f^Right:Left:Ambidextrous

^g^Measures taken at scanning session - parent report

^h^Missing data from 1 participant with conduct problems

^i^Missing data from 2 participants with conduct problems

Ninety-nine males aged 10–16 were scanned. Of these, 69 had a research diagnosis of current CP based on combined parent- and teacher- report on the Child and Adolescent Symptom Inventory (CASI-4R; Gadow and Sprafkin 2009) Conduct Disorder subscale (CASI-CD; Sprafkin and Gadow 1998). Consistent with our previous fMRI work (Sebastian et al. 2012; Viding et al. 2012), CASI-CD symptom severity scores were used to make the research diagnosis of current CP. Symptom severity cut-off scores for inclusion in the CP group were 3+ (ages 10–14) and 6+ (ages 15–16). Scores of this magnitude and above are associated with a clinical diagnosis of conduct disorder (Sprafkin and Gadow 1998). The remaining 30 were TD controls who did not differ significantly in age, IQ, handedness and SES. Of these, one TD participant was excluded due to excessive motion, and nine participants with CP were excluded due to: excessive motion (*n* = 5), scanner refusal (*n* = 3), and as it was later found that one participant did not meet CP criteria. The 60 remaining participants with CP were assigned to low (CP/LCU, *n* = 31) vs. high (CP/HCU, *n* = 29) callous-unemotional trait groups on the basis of a median split on combined parent- and teacher- reported scores on the Inventory of Callous-Unemotional Traits (ICU; Essau et al. 2006). Median ICU score within the CP group was 42: all TD controls (*n* = 29) scored below the CP group median on this measure.

For all groups, exclusion criteria included a previous diagnosis of any neurological or psychotic disorder, or a current prescription for psychiatric medication. (We later found that three participants (2 CP/LCU, 1 CP/HCU) had been medicated for ADHD symptoms during scanning. However, analyses conducted with and without these participants were very similar, and so their data were included in reported analyses). To recruit a representative sample of children with CP, common co-morbidities (ADHD, generalised anxiety disorder (GAD), major depressive episode (MDE) and substance/alcohol abuse (Connor et al. 2007) were not used as exclusion criteria, but current symptom counts were obtained (see below and Table 1).

### Psychometric and Questionnaire Measures

Participants completed the Wechsler Abbreviated Scale of Intelligence (Wechsler 1999) two-subtest version for group matching purposes, as well as Alcohol/Drug Use Disorder Identification Tests (AUDIT and DUDIT; Babor et al. 2001; Berman et al. 2005). A parent or guardian also completed the CASI-4R scales for ADHD, GAD and MDE in order to ascertain symptom counts for common co-morbidities with CP (Table 1). We did not automatically control for these variables in the main analyses reported below, as it is inappropriate to covary for variables intrinsically related to group assignment (Miller and Chapman 2001). However, for completeness we report the effects of including these covariates (as well as age and IQ) in supplementary materials (Table S1); results did not differ substantially from the main analysis.

### MRI Data Acquisition

Participants were scanned at the Birkbeck-UCL Centre for Neuroimaging using a 1.5 Tesla Siemens Avanto MRI scanner with a 32-channel head coil. A high-resolution, 3D T1-weighted structural scan was acquired using a magnetization prepared rapid gradient echo (MPRAGE) sequence. Imaging parameters were: 176 slices; slice thickness = 1 mm; gap between slices = 0.5 mm; TR = 2730 ms; TE = 3.57 ms; field of view = 256 mm x 256mm^2^; matrix size = 256 × 256; voxel size = 1 × 1 × 1 mm resolution). The scanning time was 5.5 min.

### MRI Data pre-Processing and Analysis

To quantify grey matter volume (GMV) the data were preprocessed using the VBM8 toolbox and SPM8, which provide improved segmentation and registration procedures such as the Diffeomorphic Anatomical Registration Through Exponentiated Lie Algebra (DARTEL) toolbox. As our sample was paediatric, customised tissue probability maps were created in the Montreal Neurological Institute (MNI) space for use with the VBM8 Toolbox. These were produced using the matched template approach of the Template-O-Matic Toolbox for SPM8 with each participant’s age and sex as defining variables (Wilke et al. 2008). The pre-processing steps were as follows: First, the anterior commissure was manually indicated on all structural images as the [0, 0, 0 mm] origin. Individual images were then corrected for bias-field inhomogeneities, segmented and spatially normalised (affine-only transformation) with reference to customised tissue probability maps. Segmentation accuracy was visually checked for each participant. Based on individual registered grey matter and white matter segmentations, an average DARTEL template of all 89 participants was created in MNI space (Ashburner 2007). The affine-registered grey matter and white matter segments were then warped to this average template using the high-dimensional DARTEL approach. Modulated data produced GMV, but unmodulated data were also saved to produce grey matter concentration data for subsidiary analyses. Crucially, the voxel values in the grey matter segments were only multiplied by the non-linear component of the registration to account for individual differences in brain size. Finally, grey matter segments were smoothed using a 6x6x6 mm^3^ full-width-at-half maximal Gaussian kernel to increase the signal-to-noise ratio and ensure a Gaussian distribution allowing data analysis in the general linear model (Ashburner and Friston 2005).

Analyses were performed on a voxel-by-voxel basis employing the framework of the General Linear Model within SPM8. Analyses involved a two-group comparison including combined CP group (i.e. CP/LCU and CP/HCU) versus the TD group, and a three-group comparison contrasting CP/LCU, CP/HCU and TD groups. Using an absolute threshold of 0.1, regionally-specific between-group differences in grey matter volume were assessed.

At the whole brain level, results were considered significant at the voxel level using a statistical threshold of *p* < 0.05 after Family-Wise Error (FWE) correction for multiple comparisons. However, for completeness, we also explored trends for group differences at whole brain level using a height threshold of *p* < 0.001 uncorrected (see Table S1), and an extent threshold of k = 52 voxels empirically determined according to random field theory (Hayasaka and Nichols 2004; Worsley et al. 1996). Bilateral masks were created for each of the four a priori ROIs. The masks for the amygdala, OFC, AI, and ACC were all defined using the automated anatomical labelling atlas implemented in WFU PickAtlas toolbox (Maldjian et al. 2003). Inferences in the masks were made using a statistical threshold of *p* < 0.05 after FWE-correction at the voxel level.

In regions showing group differences between CP and TD groups or between CP/LCU, CP/HCU and TD groups at *p* < 0.05 FWE, we conducted continuous analyses in SPSS version 21 within the CP group to explore the contributions of CU traits and CD symptoms to differences in GM volume in more detail. We first conducted bivariate analyses and, if significant, these were followed-up by hierarchical multiple regression to explore the contributions of unique variance associated with each variable after controlling for the other, an approach that has been used in many previous studies of childhood CP (e.g., Lozier et al. 2014; Sebastian et al. 2012). In line with previous research indicating that CU traits offer incremental utility in predicting various outcomes over and above measures of antisocial behaviour and CD (see Frick and White 2008), CASI-CD symptoms were entered first in our model, followed by CU traits in the second step. Analyses were conducted on the peak voxel showing group differences, as the peak reflects a weighted average of surrounding voxels due to smoothing.

## Results

### Total Intracranial Volume, Overall Grey Matter, White Matter Volume, and Cerebrospinal Fluid

The CP/HCU group had lower overall grey matter volume (788.86 ± 66.40 ml) in comparison to both the CP/LCU group (798.51 ml ± 58.86) and TD controls (809.06 ml ± 52.03; *F*_(2,86)_ = 5.18, *p* = 0.008; CP/HCU vs. CP/LCU (*p* = 0.016); CP/HCU vs. TD (*p* = 0.006); CP/LCU vs. TD (*p* = 0.52), but no group differences were observed for white matter volume (CP/HCU: 496.95 ml ± 55.70, CP/LCU: 522.61 ml ± 57.72, TD controls: 514.98 ml ± 55.88, *F*_(2,86)_ = 1.62, *p* = 0.205), cerebrospinal fluid (CP/HCU: 196.34 ml ± 36.20, CP/LCU: 183.64 ml ± 29.12, TD controls: 181.50 ml ± 27.44, *F*_(2,86)_ = 1.94, *p* = 0.15) or total intracranial volume (CP/HCU: 1451.64 ml ± 111.09, CP/LCU: 1504.76 ml ± 109.61, TD controls: 1505.54 ml ± 116.21, *F*_(2,86)_ = 2.22, *p* = 0.12).

### Whole Brain Analysis

**Fig. 1:**
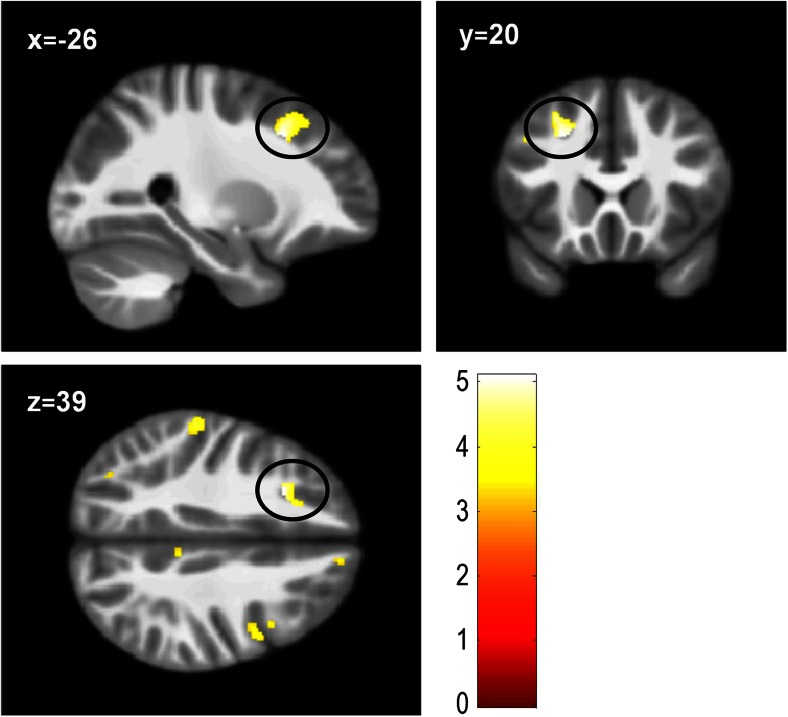
Reduced grey matter volume in the left middle frontal gyrus (peak MNI co-ordinate: x = −26, y = 20, z = 39) in the CP/HCU group (*n* = 29) compared with TD controls (*n* = 29; *p* < 0.05, FWE-corrected across the whole brain at the voxel-level). Results shown at *p* < 0.001, uncorrected (k = 543 voxels), for display purposes. Colour bar represents t-values

Comparing the CP group as a whole against the TD controls, there were no significant group differences at *p* < 0.05, whole-brain corrected. Comparing CP/HCU, CP/LCU and TD groups separately, CP/HCU exhibited reduced grey matter volume in the left middle frontal gyrus (x = −26, y = 20, z = 39; *t* = 5.1; k = 1; *p* = 0.046, FWE-corrected across the whole brain at the peak voxel level) in comparison to the TD controls (Fig. 1; in all Figures results are shown at *p* < 0.001, uncorrected for display purposes only). No other differences reached significance. Regions showing group differences at *p* < 0.001, uncorrected are reported in Table S1.

### Region of Interest Analyses

**Fig. 2:**
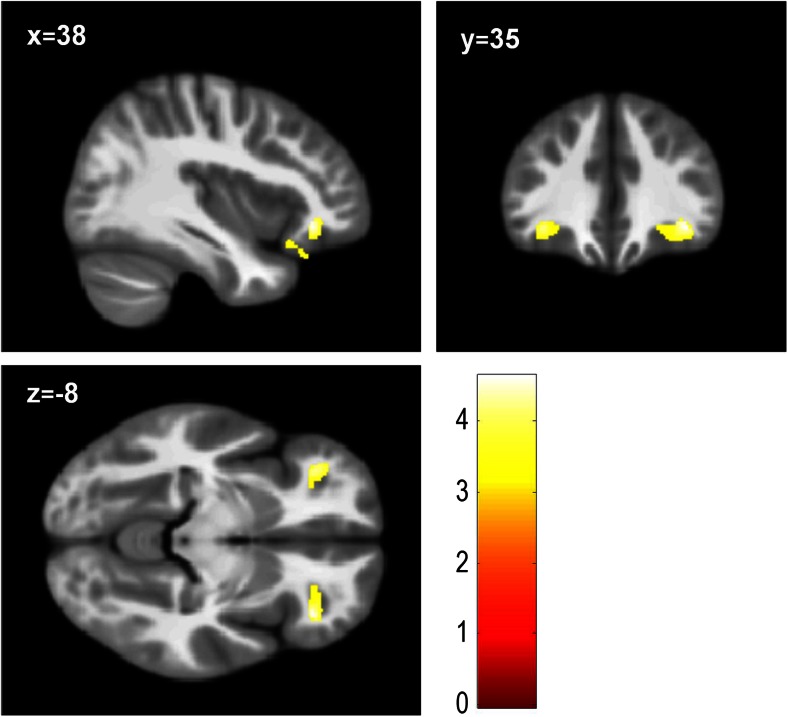
Reduced grey matter volume in bilateral orbitofrontal cortex across CP groups (*n* = 60) compared with TD controls (*n* = 29; *ps* < 0.05, FWE-SVC). Peak MNI co-ordinates: right: x = 38, y = 35, z = −8; left: x = −36, y = 36, z = −8. Results in the right (k = 462 voxels) and left (k = 285 voxels) orbitofrontal cortex are shown at *p* < 0.001, uncorrected, for display purposes. Colour bar represents t-values

**Fig. 3:**
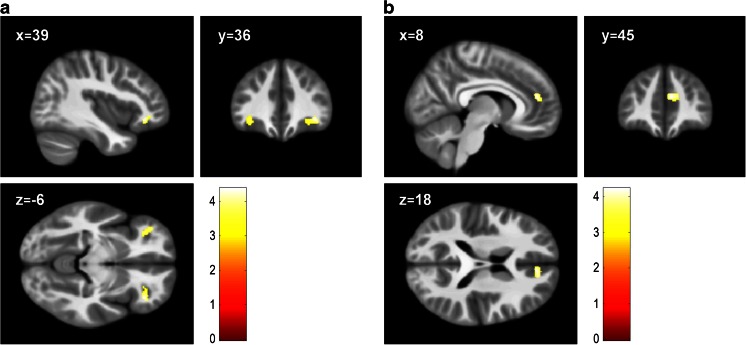
Reduced grey matter volume in the CP/HCU group (*n* = 29) compared with TD controls (*n* = 29; *ps* < 0.05, FWE-SVC) in **a)** bilateral orbitofrontal cortex (peak MNI co-ordinates: right: x = 39, y = 36, z = −8; left: x = −38, y = 44, z = −6) and **b)** right anterior cingulate cortex (peak: x = 8, y = 45, z = 18). Result in the right (k = 194 voxels) and left (k = 290 voxels) orbitofrontal cortex and right anterior cingulate cortex (k = 173 voxels) are shown at *p* < 0.001, uncorrected, for display purposes. Colour bar represents *t*-values

**Fig. 4:**
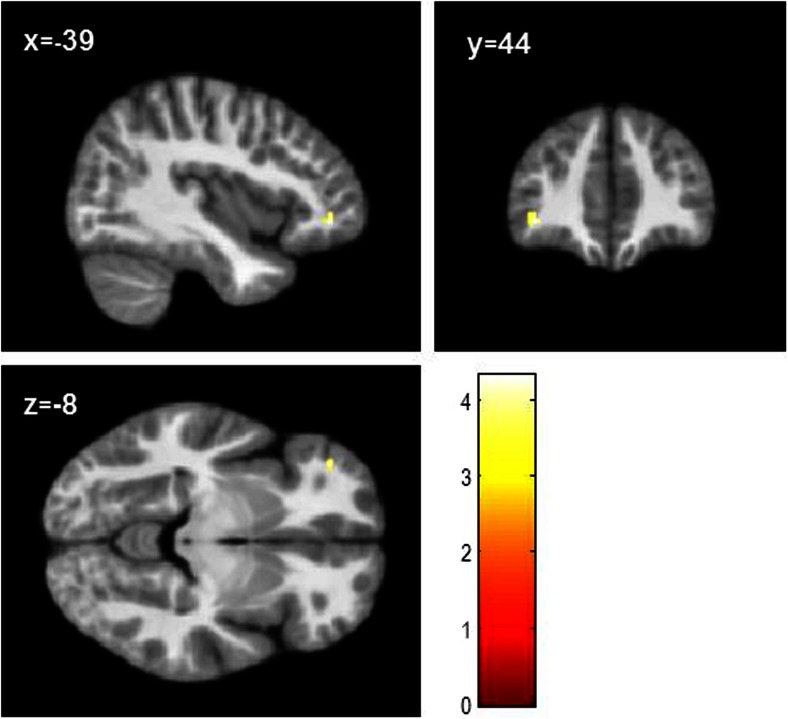
Left orbitofrontal cortex also showed grey matter volume reduction in the CP/HCU group (*n* = 29) compared with the CP/LCU group (*n* = 31; *p* < 0.05, FWE-SVC; peak: x = −39, y = 44, z = −6). Results shown at *p* < 0.001, uncorrected (k = 39 voxels), for display purposes. Colour bar represents *t*-values

In comparison with TD controls, children with CP had reduced grey matter in the OFC bilaterally [Right: x = 38, y = 35, z = −8; *t* = 4.63; k = 22; *p* = 0.01 FWE-small volume corrected (SVC); Left: x = −36, y = 36, z = −8; *t* = 4.20; k = 4; *p* = 0.041 FWE-SVC; (Fig. 2)]. However, looking at CP/HCU and CP/LCU separately, only the CP/HCU group exhibited reduced grey matter in bilateral OFC relative to TD controls [Right: x = 39, y = 36, z = −8; *t* = 4.38; k = 8; *p* = 0.024 FWE-SVC; Left: x = −38, y = 44, z = −6; *t* = 4.39; k = 4; *p* = 0.023 FWE-SVC; (Fig. 3a; Fig. S1a and S1b)]. In addition, children with CP/HCU also showed reduced grey matter in right ACC compared with TD controls [x = 8, y = 45, z = 18; *t* = 4.24; k = 42; *p* = 0.014 FWE-SVC; (Fig. 3b and Fig. S1c)]. Finally, a direct comparison between the two CP groups revealed that, relative to the CP/LCU group, children with CP/HCU showed reduced grey matter in left OFC [x = −39, y = 44, z = −6; *t* = 4.31; k = 1; *p* = 0.029 FWE-SVC; (Fig. 4)]. There were no significant differences between CP/LCU and TD controls in any ROI at FWE-corrected levels. Results for analyses of grey matter concentration closely matched those obtained with grey matter volume metrics.

### Continuous Analyses: CU traits and CD symptoms

In the left OFC where the CP/HCU group showed reduced GMV compared to the CP/LCU group, GMV was significantly negatively correlated with CU traits (zero-order correlation *r*_(60)_ = −0.36; *p* = 0.004), but only marginally negatively correlated with CD symptoms (*r*_(60)_ = −0.22; *p* = 0.09). A hierarchical multiple regression analysis in which CD symptoms were entered in a first step and CU traits were entered in a second step was conducted to explore contributions of these variables to variance in left OFC volume. In line with the bivariate correlations above, results indicated that the model with CD symptoms entered first was not statistically significant (*F*_(1,58)_ = 2.92, *p* = 0.09, *R*^2^ = 0.048). However, the model including both CD symptoms and CU traits was significant (*F*_(2,57)_ = 4.35, *p* = 0.017, *R*^2^ = 0.13). The *R*^2^ change after including CU traits in the model was also significant (*F*_(1,57)_ = 5.56, *p* = 0.022). Looking at the unique contributions of each variable in the final model, CU traits significantly predicted left OFC volume (*β* = −0.34, *t*_(59)_ = −2.36, *p* = 0.022), while CD symptoms did not (*β* = −0.036, *t*_(59)_ = −0.25, *p* = 0.80).

There were no statistically significant zero-order correlations (all *ps* > 0.32) with GMV in the other regions showing group difference in the SPM analyses at *p* < 0.05 FWE-corrected.

## Discussion

The current study used VBM to compare grey matter volumes in subgroups of children with CP and high vs. low levels of CU traits, and TD controls. We used a large sample (N = 89) together with state-of-the-art anatomical registration methods to maximise statistical power and accuracy of data pre-processing. In whole-brain analyses, we found evidence for reduced GM volume in CP/HCU relative to TD controls in the left middle frontal gyrus. Comparing groups in a priori ROIs, we found that reduced bilateral OFC GM volume in the CP group as a whole relative to TDs was largely driven by the CP/HCU group. Directly comparing all three groups, the CP/HCU group showed reduced bilateral OFC volume relative to TD controls, and reduced left OFC volume relative to both TD controls and CP/LCU. In contrast, there were no differences between CP/LCU and TD groups at FWE-corrected levels (although it is worth noting that a small cluster showed reduced right OFC volume in CP/LCU compared with TD controls at *p* < 0.001 uncorrected; Table S1). Continuous analyses within the CP group showed that these group differences were likely driven by CU traits as opposed to levels of CD symptoms. A largely comparable pattern of group differences was also seen in right ACC, again with reduced GM volume in CP/HCU relative to TD controls and no difference between CP/LCU and controls, even at uncorrected levels. However, CP/HCU and CP/LCU groups did not differ from each other in this region, even at uncorrected levels.

Taken together, the pattern of results supports several previous studies which have found GM reduction in children and adolescents with CP relative to TD controls in regions including OFC and ACC (Cope et al. 2014a; Fahim et al. 2011; Fairchild et al. 2011; Huebner et al. 2008). Our study extends these findings to suggest that GM reduction in some of the regions identified in these studies may in fact be primarily attributable to those children in whom CP co-occur with high levels of CU traits, as opposed to being associated with CP in general. This finding is in line with a study by Ermer et al. (2013) which found negative associations between OFC GM volume and psychopathic traits in a sample of incarcerated male adolescents. However, we extend this work by showing that differences between CP/HCU and TD controls, specifically in left OFC and right ACC, did not characterise the CP/LCU group. Indeed in left OFC, the CP/LCU group showed significantly increased GM volume relative to CP/HCU, i.e. the same pattern as TD controls. These results show GM volume in these two regions (particularly in left OFC), may differentiate these two subgroups of children with CP. Overall, considering the results at corrected and uncorrected levels (Table S1), the CP/HCU group showed more extensive grey matter reductions than the CP/LCU group when compared to the TD groups as evidenced by additional reductions in grey matter volumes in the ACC (FWE-corrected) and in the anterior insula (uncorrected), among other regions, which were not observed in the CP/LCU group, even at uncorrected levels. These results tentatively suggest that, compared to TD controls, the two CP groups might be characterized by distinct grey matter differences in regions central to decision-making, empathy and emotion regulation. The more widespread reduction in the CP/HCU group might thus partly explain their different behavioural and neurobiological profiles (Frick and Viding 2009).

These results are consistent with evidence suggesting that CP/HCU in childhood and psychopathic traits in adulthood are associated with atypical OFC and ACC function (e.g., (Anderson and Kiehl 2012; Blair 2013). In OFC, Finger et al. (2011) found reduced fMRI responses in a network of regions including OFC during a reinforcement learning task in youths with CP and elevated psychopathic traits; while Marsh et al. (2011) found reduced OFC-amygdala connectivity in a similar sample during a moral judgment task. Behavioural work has also shown subtle impairments on OFC-dependent tasks such as reversal learning (e.g., Budhani and Blair 2005). In ACC, a recent study using a partially overlapping sample to that reported here found reduced responses in children with CP when viewing pictures of others in pain (Lockwood et al. 2013); moreover, activity in this region was negatively associated with levels of CU traits. A similar result in ACC was also found by Marsh et al. (2013). While we cannot equate functional hypo-reactivity and reduced GM volume, the current data are consistent with theories suggesting that atypical neural function in regions underlying emotional processing and reinforcement learning contributes to CP/HCU (e.g., Anderson and Kiehl 2012; Blair 2013). Future studies could use multimodal imaging to explore relationships between structural and functional measures in children with CP.

Of the four ROIs, amygdala and AI did not show group differences at FWE-corrected levels (although the CP group as a whole showed reduced right AI volume at *p* < 0.001 uncorrected; Table S1). This was somewhat surprising, since previous studies have found reduced volume of these regions in children and adolescents with CP (Fairchild et al. 2011; Sterzer et al. 2007), while several fMRI studies (including three based on a subset of the participants included in the current study) have found evidence for amygdala and/or AI hypoactivity during emotional processing in CP/HCU (Jones et al. 2009; Lockwood et al. 2013; Marsh et al. 2008; Sebastian et al. 2012; Viding et al. 2012). There is therefore strong evidence across imaging modalities for atypical amygdala and AI function in this group. However, not all sMRI studies in children with CP have found GM reductions in these regions. For example De Brito et al. (2009) and Fahim et al. (2011) did not find any group difference in amygdala volume, while Huebner et al. (2008) did not observe reduced volume of the AI. Additionally, in the largest VBM study to date in this area (Ermer et al. 2013), only weak relationships were found between amygdala volume and psychopathic traits, while no relationships were observed between AI GM volume and psychopathic traits. Atypical amygdala and AI function therefore appear to be more robustly associated with CU traits than does atypical structure in these regions.

At the whole brain level, reduced GM volume was also seen in left middle frontal gyrus in CP/HCU relative to controls. While this region was not hypothesised to show group differences a priori, the result survived FWE-correction across the whole brain and so may represent an additional marker for CP/HCU. Studies reporting activation within 6 mm of the peak within this cluster (based on Neurosynth location data, http://www.neurosynth.org/locations) have typically implicated this region in higher cognitive processes such as strategy use (Bor and Owen 2007), and context-dependent episodic retrieval (King et al. 2005). However, it has also been shown to contribute to emotional processes, for example dynamic (vs. static) emotional face perception (Trautmann et al. 2009). While it is too early to conclude that this region is implicated in the pathophysiology of CP/HCU, our finding could nonetheless be useful in motivating further investigations of this region’s structure and function in this group.

The pattern of GM reduction reported in the current study is not in line with our previous finding of increased GM concentration in OFC and ACC in CP/HCU (De Brito et al. 2009). In our view, the most likely explanation is the differing ages of the samples. The current sample were considerably older (mean age = 14 years, 0 months; age range: 10.2–16.9), whilst De Brito et al. (2009) studied a younger group (mean age = 11 years, 7 months; age range: 10.0–13.3). These previous results were interpreted as reflecting delayed cortical maturation in the CP/HCU sample relative to the typically observed pattern of GM reduction with age (Gogtay et al. 2004; Shaw et al. 2008). Delayed cortical maturation is common to several developmental disorders (Shaw et al. 2010). However, developmental trajectories may look different at later points in the lifespan; for example in children with ADHD, (Shaw et al. 2012) found a delay in the age at which childhood increase in cortical thickness gives way to cortical thinning, similar to the delayed reduction in GM concentration seen in De Brito et al. (2009). However, a longitudinal study including adults with ADHD symptoms found that by adulthood, symptom severity was associated with reduced GM thickness (Shaw et al. 2013). The relatively young age of participants in De Brito et al. (2009) may therefore contribute to the differing pattern of results seen relative to the current study. However, longitudinal investigation of GM trajectories in CP is required to test this hypothesis directly.

While the current study has several methodological strengths, including a large sample size and the use of age- and gender-specific templates together with DARTEL registration, it is also worth noting some limitations. First, as with all previous sMRI studies of CP, given the cross-sectional design we are unable to infer whether neural differences are a cause or a consequence of the group differences observed. Second, VBM provides a composite measure of surface area and cortical thickness, and cannot provide a fine-grained analysis of specific GM metrics that may be driving the observed group differences (Raznahan et al. 2011). Further, given evidence that antisocial behaviour is more strongly heritable in children with CP/HCU than CP/LCU (Viding et al. 2005; Viding et al. 2008) and that surface area and cortical thickness are highly heritable, yet genetically unrelated (Panizzon et al. 2009), future studies should directly compare youths with CP/HCU and CP/LCU using those metrics. Additionally, while our groups did not differ significantly in age, IQ, ethnicity, handedness and SES, they differed on several comorbid psychopathology variables including ADHD, depression, anxiety and alcohol use. Therefore it could be argued that the group differences obtained are not specific to CP/HCU, but reflect a more severe profile of general psychopathology and a lower IQ. A related argument is that group differences resulted from differences in the severity of conduct disorder symptoms rather than CU traits. However, we think these alternative explanations are unlikely. Results from symptom and IQ covariate analyses were very similar to the main findings (see footnote Table S1). Importantly, while CP/HCU and CP/LCU groups differed on both CU traits and CD symptoms (which are typically modestly correlated, and were correlated at *r* = 0.53 in the current sample), only CU traits correlated negatively with left OFC volume. When the unique contribution of CU traits and CD symptoms to differences in left OFC was examined (after controlling for the other variable), the negative relationship with CU traits was strengthened, while the trend-level contribution of CD symptoms was reduced to near zero. Moreover, CU traits significantly improved the ability of our regression model to predict left OFC volume relative to CD symptoms alone. We took the decision to recruit a representative sample of children with CP as opposed to recruiting a fully matched sample which would likely have been unrepresentative in unpredictable ways. It is also worth noting that there is a strong theoretical basis to the idea that CU traits are a contributing explanatory factor for more severe conduct problem symptoms (Frick and Viding 2009); hence the focus on CU traits in the current study.

Overall, we replicate and extend previous studies showing a reduction in grey matter volume in children with CP in OFC and ACC: key regions of interest associated with emotional processing and reinforcement learning. Reductions in left OFC and right ACC were restricted to a subgroup of children with CP characterised by high levels of CU traits: reduced GM volume here therefore seems to characterise the CP/HCU, but not the children with CP/LCU, who also exhibit conduct disturbance but have differing genetic and neurocognitive vulnerabilities. To our knowledge, no previous study has compared CP/HCU and CP/LCU groups directly on tasks tapping the functions of these regions, so this may be a fruitful avenue of research. More generally, the present findings strengthen the case that it is important to take into account levels of CU traits in the diagnosis and treatment of children with CP.

## Electronic supplementary material

Online Supplementary Figures S1a-c: Grey matter volume values at peak voxel for the TD, CP/LCU and CP/HCU groups in (a) right orbitofrontal cortex (x = 39, y = 36, z = −8), (b) the left orbitofrontal cortex (x = −38, y = 44, z = −6), and (c) right anterior cingulate cortex (x = 8, y = 45, z = 18). (GIF 164 kb)

High resolution image (TIFF 86 kb)

ESM 2 (XLSX 20 kb)
