# Supplementary figures and images for: Grey Matter Volumes in Children with Conduct Problems and Varying Levels of Callous-Unemotional Traits
Source: J Abnorm Child Psychol. 2015 Sep 14;44:639–49. doi: 10.1007/s10802-015-0073-0 (PMC4830891; doi:10.1007/s10802-015-0073-0)

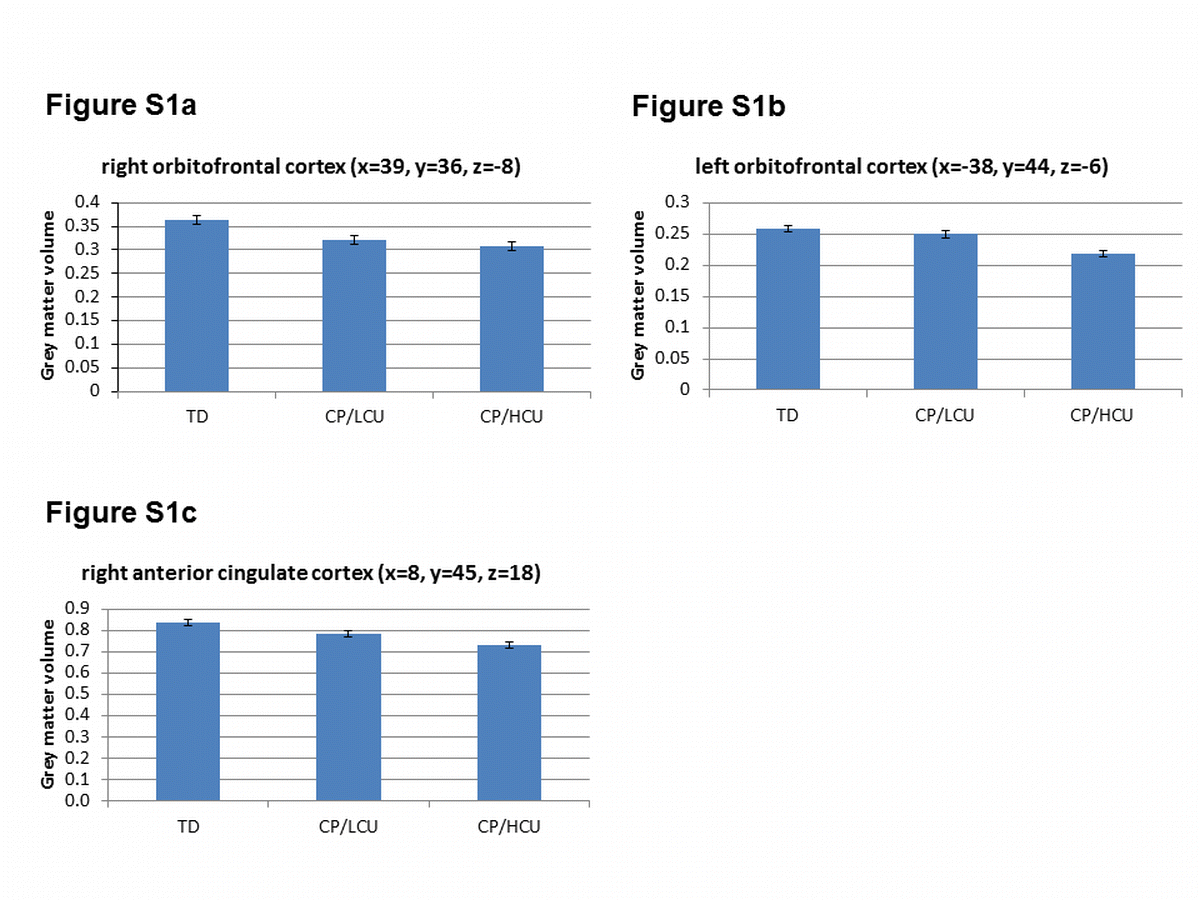

Supplement: Supplementary file 1 — Grey matter volume values at peak voxel for the TD, CP/LCU and CP/HCU groups in (a) right orbitofrontal cortex (x = 39, y = 36, z = −8), (b) the left orbitofrontal cortex (x = −38, y = 44, z = −6), and (c) right anterior cingulate cortex (x = 8, y = 45, z = 18). (GIF 164 kb) [file 10802_2015_73_Fig5_ESM.gif]

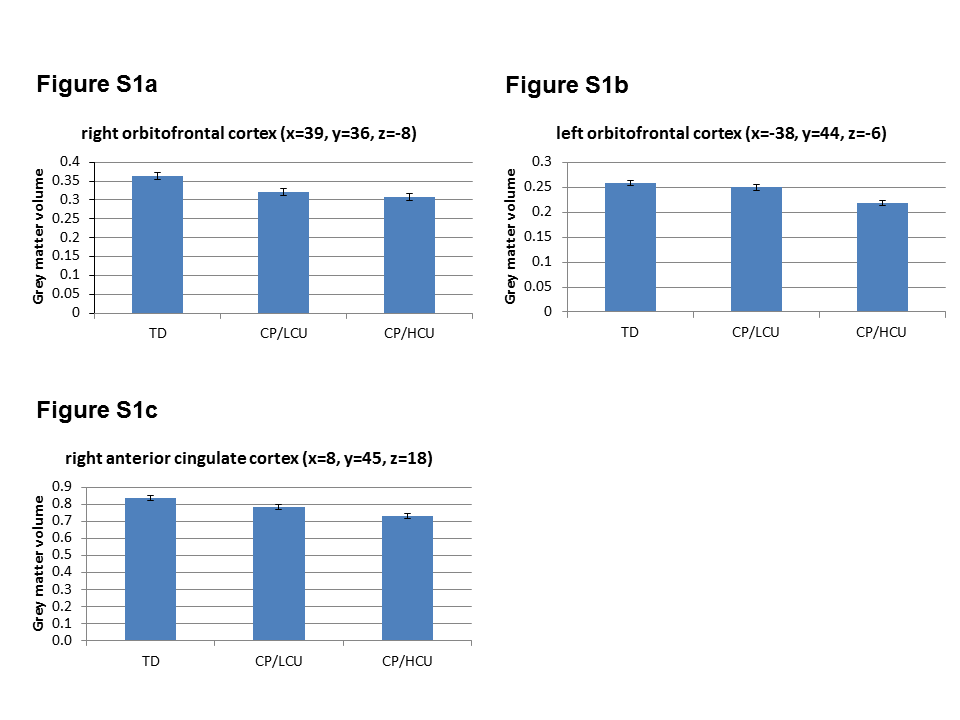

Supplement: Supplementary file 2 — High resolution image (TIFF 86 kb) [file 10802_2015_73_MOESM1_ESM.tif]
